# Phosphoproteomics Identifies Oncogenic Ras Signaling Targets and Their Involvement in Lung Adenocarcinomas

**DOI:** 10.1371/journal.pone.0020199

**Published:** 2011-05-26

**Authors:** Putty-Reddy Sudhir, Chia-Lang Hsu, Mei-Jung Wang, Yi-Ting Wang, Yu-Ju Chen, Ting-Yi Sung, Wen-Lian Hsu, Ueng-Cheng Yang, Jeou-Yuan Chen

**Affiliations:** 1 Institute of Biomedical Sciences, Academia Sinica, Taipei, Taiwan; 2 Institute of Biomedical Informatics, National Yang-Ming University, Taipei, Taiwan; 3 Institute of Chemistry, Academia Sinica, Taipei, Taiwan; 4 Institute of Information Sciences, Academia Sinica, Taipei, Taiwan; 5 Institute of Genome Sciences, National Yang-Ming University, Taipei, Taiwan; 6 Department of Life Sciences, National Yang-Ming University, Taipei, Taiwan; Cleveland Clinic, United States of America

## Abstract

**Background:**

Ras is frequently mutated in a variety of human cancers, including lung cancer, leading to constitutive activation of MAPK signaling. Despite decades of research focused on the Ras oncogene, Ras-targeted phosphorylation events and signaling pathways have not been described on a proteome-wide scale.

**Methodology/Principal Findings:**

By functional phosphoproteomics, we studied the molecular mechanics of oncogenic Ras signaling using a pathway-based approach. We identified Ras-regulated phosphorylation events (n = 77) using label-free comparative proteomics analysis of immortalized human bronchial epithelial cells with and without the expression of oncogenic Ras. Many were newly identified as potential targets of the Ras signaling pathway. A majority (∼60%) of the Ras-targeted events consisted of a [pSer/Thr]-Pro motif, indicating the involvement of proline-directed kinases. By integrating the phosphorylated signatures into the Pathway Interaction Database, we further inferred Ras-regulated pathways, including MAPK signaling and other novel cascades, in governing diverse functions such as gene expression, apoptosis, cell growth, and RNA processing. Comparisons of Ras-regulated phosphorylation events, pathways, and related kinases in lung cancer-derived cells supported a role of oncogenic Ras signaling in lung adenocarcinoma A549 and H322 cells, but not in large cell carcinoma H1299 cells.

**Conclusions/Significance:**

This study reveals phosphorylation events, signaling networks, and molecular functions that are regulated by oncogenic Ras. The results observed in this study may aid to extend our knowledge on Ras signaling in lung cancer.

## Introduction

Constitutive activation of Ras-mediated signaling and its downstream components, such as MAP family kinases, play an important role in the pathogenesis of human cancers 1,2]. Lung cancer represents a good choice for investigating the molecular mechanisms of Ras-mediated MAPK signaling, because the activation of Ras oncogenes by mutation or amplification has been reported most frequently in lung cancer 1,3]. Among the post-translational modifications, reversible protein phosphorylation is a dominant regulatory mechanism involved in the oncogenic signaling process. Simultaneous identification and quantification of phosphorylation events induced by oncogenic signaling not only would provide insight into signaling mechanisms but also are essential for understanding the molecular determinants of disease progression. Despite decades of intensive analysis of the Ras family of proto-oncogenes (*HRAS*, *NRAS,* and *KRAS*) and corresponding biological processes 2,4], the downstream phosphorylation targets and pathways regulated by oncogenic Ras-mediated signaling remain to be elucidated in lung cancer.

Apart from traditional molecular biological approaches, alternative strategies are needed to identify downstream phosphorylation targets and regulated pathways of oncogenic signals. Presently, quantitative phosphoproteomics is being widely used and is emerging as a key technology in signal transduction research 5]. The *in vitro* and *in vivo* incorporation of stable isotopes into samples has been introduced and is extensively used 6,7]. However, the isotopic labeling-based quantitation limits the analysis of a large number of samples in a single experiment. Hence, label-free quantitation methods have gained more popularity in recent years 8]. To gain further information on Ras-regulated cellular processes, we conducted a pathway-based investigation to evaluate Ras activity. We identified Ras-mediated phosphorylation events in immortalized human bronchial epithelial cells (HBECs) using IDEAL-Q (ID-based elution time prediction by fragmental regression)-based quantitation proteomics, followed by computational methods to infer Ras-mediated signaling pathways and molecular functions. Furthermore, the interpretation of Ras-mediated phosphorylation targets and related pathways allowed us to demonstrate the involvement of Ras-mediated signaling in lung adenocarcinoma (AD), but not in large cell carcinoma (LCC). Our findings on the phosphorylation events, kinomes, and pathways regulated by oncogenic Ras and differential activation of Ras downstream signaling in lung AD and LCC could serve as a basis for future investigations elucidating the molecular mechanisms involved in the pathogenesis of human cancers.

## Methods

### Cell culture

Cdk4 (cyclin-dependent kinase 4)/hTERT (human telomerase reverse transcriptase)-immortalized human bronchial epithelial cells (HBEC3-KT or 3KT) and K-RAS^V12^-transformed HBEC3-KT cells (3KTR) were maintained in K-CFM medium containing 50 µg/mL bovine pituitary extract (BPE) and 5 ng/mL EGF under 5% CO_2_ and 37°C, as previously described 9,10]. These cells were the kind gift of Dr. John D. Minna (University of Southwestern Medical Center, Dallas, TX, USA). The human non-small-cell lung cancer (NSCLC) H1299, H322, and A549 cell lines were purchased from the American Type Culture Collection. H1299 and H322 cells were grown in RPMI medium supplemented with 10% fetal bovine serum and 1% antibiotics under tissue culture conditions. A549 cells were grown under similar conditions in DMEM supplemented with 10% fetal bovine serum and 1% antibiotics.

### Knockdown experiments, lysis, western blot analysis, tryptic digestion, and phosphopeptide enrichment

The lentivirus-based knockdown approach has been previously described [11]. The pLKO.1-short hairpin RNA (shRNA) plasmids encoding an shRNAs with sequences targeting the firefly *luciferase* and with sequences targeting human KRAS (*sh1*: 5′-CTATGGTCCTAGTAGGAAAT-3′; and *sh2*: 5′-GAGGGCTTTCTTTGTGTATTT-3′) purchased from the National RNAi Core Facility, Taiwan, were introduced into HEK293T cells with lentiviral packaging vectors pMD.G and pCMV 8.91. Viruses were collected from the medium 60 h after transfection. For knockdown experiments, cells were infected with the collected viruses over 24 h in the presence of Polybrene (at 3 MOI for H322 and 5 MOI for A549 and H1299). Cells were cultured in designated medium for 24 hr prior to lysis. To prepare cell lysates, cells were grown to 85% confluence and subjected to lysis using modified RIPA buffer containing 0.5% NP40 (Igepal CA-630), 300 mM NaCl, 1 mM EDTA, 0.5 mM DTT, 1 mM NaVO_3_, 10 mM NaF, and a cocktail of protease inhibitors (Roche, Indianapolis, IN, USA). After centrifugation at 15,000× *g* for 5 min, the cell lysates were collected. Western blotting was performed as previously described [11]. Tube-gel digestion was performed as previously described [12] with modifications. Phosphopeptides were purified using a revised one-step IMAC as previously described [13]. For more details, see Methods S1.

### Mass spectrometry analysis and label-free quantitation

Purified phosphopeptide samples were desalted and analyzed by LC-MS/MS (Waters Q-Tof Premier^TM^; Waters Corp., Milford, MA, USA). The MS was operated in ESI positive V mode with a resolving power of 10,000. Data were acquired via Data Directed Analysis (DDA). The method included a full sequential MS scan (m/z 400–1600, 0.6 s) and 3 MS/MS (m/z 100–1990, 1.2 s per scan) of the three most intense ions present in the full-scan mass spectrum. The details of the MS analysis, peptide identification, and label-free quantitation using the IDEAL-Q algorithm [14] are available in Methods S1.

### Calculation of pathway activity

Based on the report of Lee et al. [15], we developed a method to infer the activity of individual signaling pathways by integrating the levels of related phosphoprotein signatures determined by proteomics analysis. For each pathway, the representative values of phosphoproteins were summed, and the mean (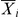) and standard deviation (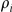) for each class (e.g., 3KT and 3KTR) were defined. The pathway activity (*a_p_*) was derived using *t*-test statistics to discriminate between two classes and formulated.

### Pathway functional clustering

Pathways were clustered based on their functional similarity. The functional similarity was computed for each pathway pair based on the Gene Ontology (GO) annotations of proteins in each pathway. The details of pathway functional clustering are available in Methods S1 [16]–[18].

## Results

### Phosphopeptide profiling of HBECs and NSCLC cells

**Figure 1:**
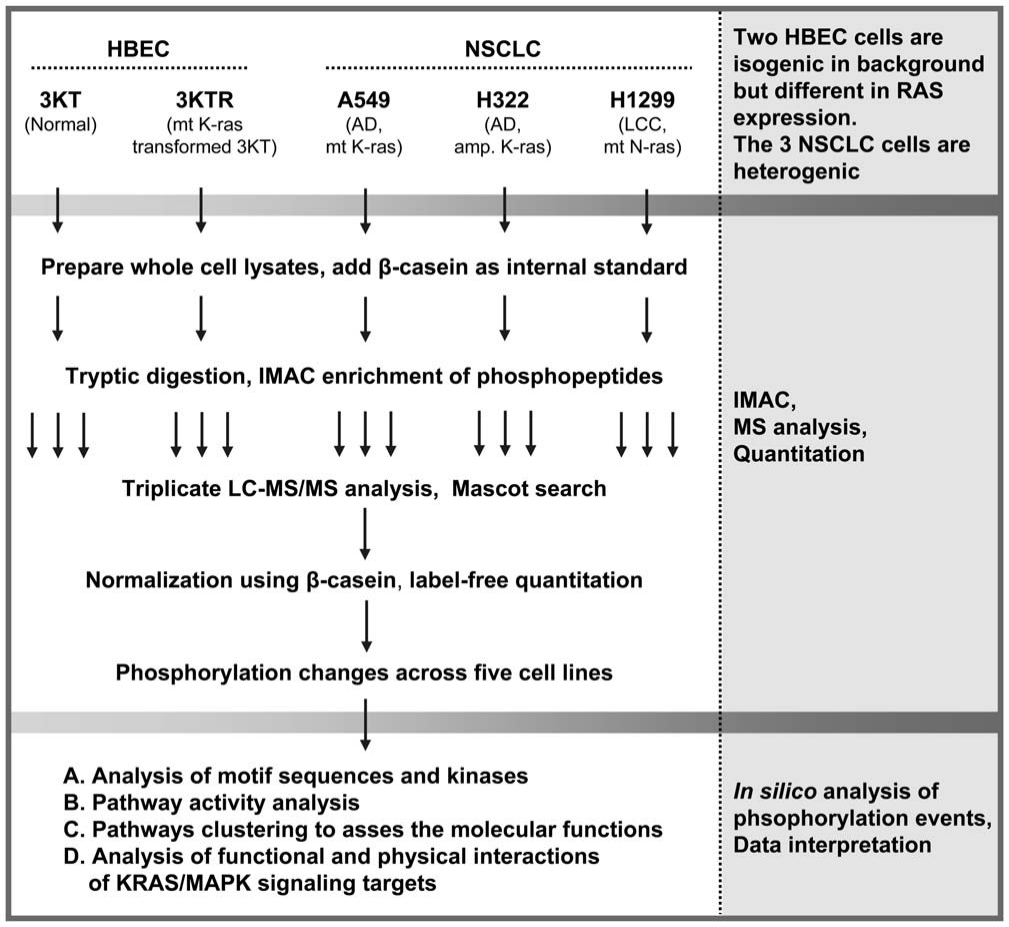
Schematic of the experimental design. Whole cell extracts were prepared from Cdk4/hTERT-immortalized human bronchial epithelial cells (HBEC3-KT or 3KT), KRAS^V12^-transformed 3KT (3KTR), and NSCLC A549, H322, and H1299 cells, then mixed with β-casein as an internal standard, and subjected to trypsin digestion. Phosphopeptides were enriched from each cell extract using IMAC and analyzed three times by LC-MS/MS. Peptides were identified by Mascot search, and phosphorylation changes were quantified in pairs across the five cell lines using the IDEAL-Q label-free system. The interpretation of phosphoproteomics data was performed by means of *in silico* analysis. Mt, mutant; AD, adenocarcinoma; LCC, large cell carcinoma; IMAC, immobilized metal affinity chromatography.

To study Ras-mediated downstream phosphorylation events and Ras involvement in lung cancer, Cdk4/hTERT-immortalized human bronchial epithelial cells (HBEC3-KT or 3KT) and KRAS^V12^-transformed 3KT (3KTR) cells were subjected to quantitative phosphoproteomics analysis. Three non-small cell lung cancer (NSCLC)-derived cell lines, A549 (adenocarcinoma, AD), H322 (AD), and H1299 (large cell carcinoma, LCC), were also studied. The flowchart in Figure 1 outlines the strategy followed in this study. The phosphopeptides enriched from each cell lysate were analyzed three times by LC-MS/MS. A total of 1645 phosphorylated peptides derived from 756 proteins were identified across the cell lines. More than 80% of the phosphopeptides were identified in triplicate runs of each cell line (Figure S1A). The abundances of phosphopeptides were shown less than 20% coefficient of variation (CV) in triplicate runs (Figure S1B). For quantitation, we first examined the abundance of the co-enriched phosphopeptide (^49^FQpSEEQQQTEDELQDK^63^) from β-casein, an internal standard, and it was consistent in the triplicate runs of each cell lysate and comparable with those from other cell lysates, demonstrating the efficiency of tryptic digestion and the IMAC enrichment process (Figure S1C). Phosphopeptides were then normalized using an internal standard and compared between the transformed and normal cell lines (3KTR, A549, H322, or H1299 vs. 3KT) and among the three NSCLC cell lines (H1299 vs. A549 or H322, and A549 vs. H322) by means of the label-free IDEAL-Q quantitation system. IDEAL-Q, a fully automated tool, performs normalization and quantitation of comparable peptides between parallel runs of unlimited samples [14]. The quantitative comparison and normalization performed in the case of 3KTR vs. 3KT are shown in Figure S1D and S1E.

**Figure 2:**
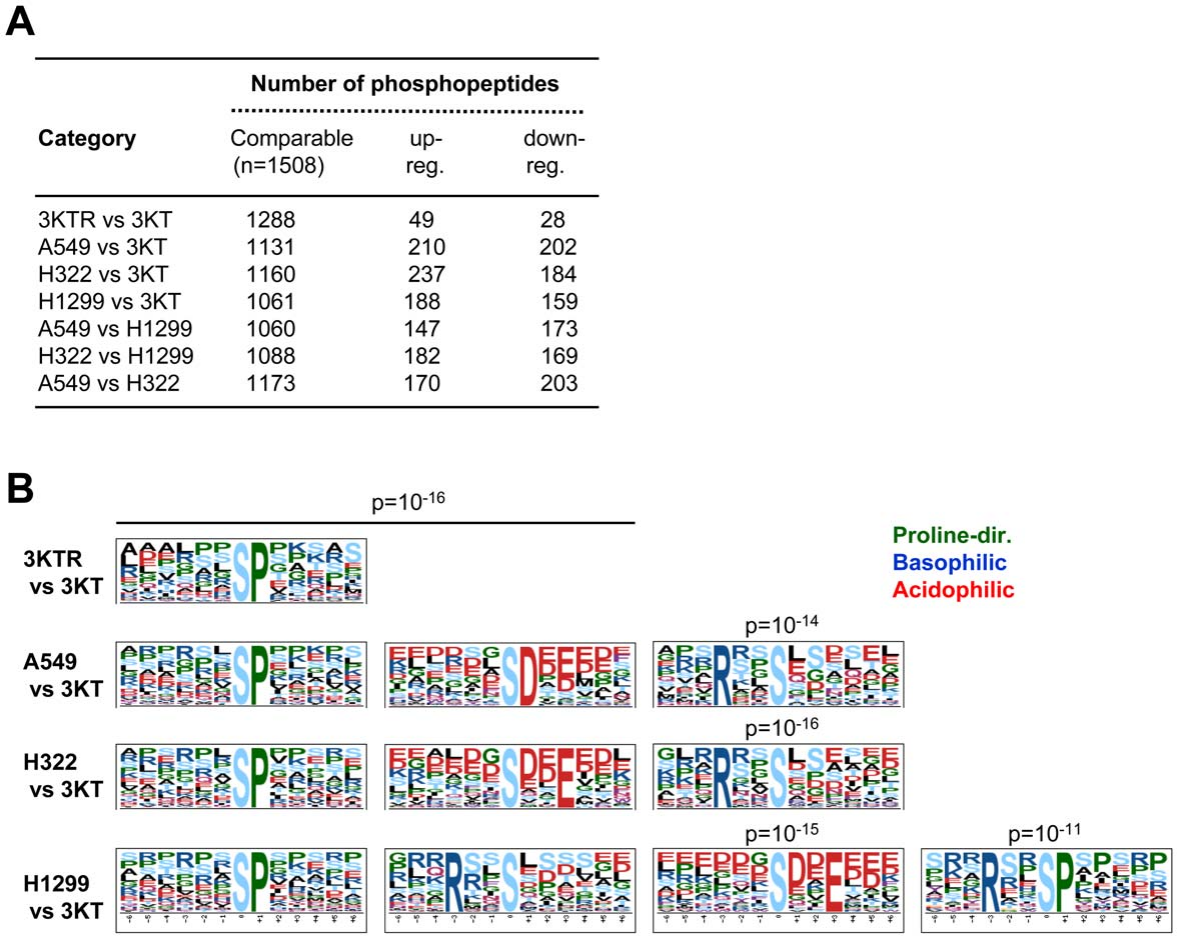
Motifs or consensus sequences of regulated phosphorylation events identified in Ras-transformed HBECs and NSCLC cells. (A) The numbers of comparable phosphopeptides subjected to quantitative proteomics analysis and the upregulated and downregulated phosphopeptides identified in each pair of cell lines are shown. (B) Representative motif sequences were extracted from the regulated phosphopeptides using motif-x. The motifs with significance of p<10^−10^ are shown.

Of the phosphopeptides identified (n = 1645), 1508 peptides from 696 proteins were quantified based on their concurrent detectability in the individual pairs of cell lines to be compared (Figure 2A and Table S1). For any pair of cell lines, 70.3 to 85.4% of the phosphopeptides were quantified, and for any cell line, >95% of the phosphopeptides were quantified. In the case of 3KTR vs. 3KT, 1288 phosphopeptides were quantified, and most (94%) displayed a relative expression ratio within twofold, consistent with the isogenic background of these two cell lines. In the comparisons of non-isogenic cells (A549 or H322 or H1299 vs. 3KT; A549 or H322 vs. H1299; and A549 vs. H322), more regulated phosphorylation events (30.2–36.4%) were identified. The regulated phosphosites were further sorted using motif-x [19]. This resulted in the identification of a proline-directed ([pS]-P) motif as an over-represented species in the regulated phosphopeptides in Ras-transformed HBECs and NSCLC cells. Additional basophilic (R-X-X-[pS]) and acidophilic ([pS]-[D/X]-X-[E/X]) motifs were also found in the NSCLC cells (Figure 2B).

### Defined subsets of kinases are differentially activated in Ras-transformed HBECs and NSCLC cells

**Figure 3:**
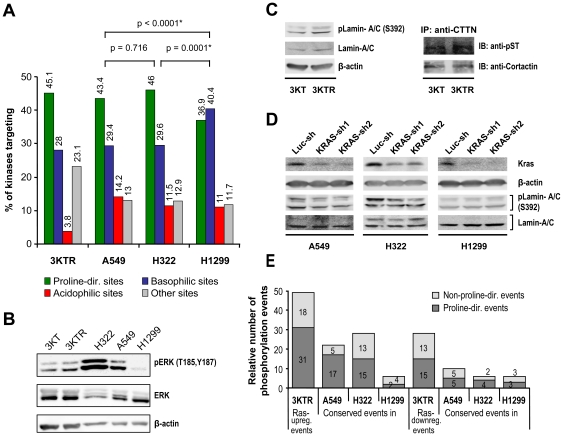
Characterization of the differentially phosphorylated events in Ras-transformed HBECs and NSCLC cells. (A) The kinases targeting regulated phosphosites were derived using NetworKIN analysis. According to their target sequences, the upstream kinases were grouped into subsets of proline-directed, basophilic, acidophilic, and other kinases. Their frequencies are shown for each cell line. (B) Western blot analysis of ERK activation in HBECs and NSCLC cells. (C) Phosphorylation of lamin-A/C and cortactin in HBECs. Western blot analysis was performed to examine the phosphorylation of lamin-A/C at S392 using site-specific antibody. Phosphorylation of cortactin (CTTN) was determined by immunoprecipitation of cortactin in cell lysates using anti-cortactin antibody followed by immunoblot analysis using antibody specific for phosphorylated Ser/Thr. (D) Western blot analysis of lamin-A/C phosphorylation in KRAS knockdown NSCLC cell lines. Lung cancer A549, H322 and H1299 cells were infected with Lentivirus harboring shRNAs targeting at *luciferase* or KRAS overnight. Two individual shRNAs targeting at KRAS were employed. Cells were grown in serum-free medium for an additional 24 hrs prior to western blot analysis of designated proteins. The status of lamin-A/C pS392 was detected by site-specific antibody. β-actin was used as protein loading control. (E) Ras-regulated phosphorylation events observed in Ras-transformed HBECs and NSCLC cells. The Ras-regulated phosphorylation events, including 49 upregulated and 28 downregulated events, identified in Ras-transformed HBECs were classified as proline-directed and non-proline-directed phosphosites according to their consensus sequences. The conservation of Ras-regulated phosphorylation events in NSCLC A549, H322, and H1299 cells is shown.

Given that the regulated phosphopeptides consisted of motif sequences with distinctive features, we further explored the potential upstream kinases accountable for the regulated phosphorylation events by NetworKIN analysis. The NetworKIN algorithm predicts *in vivo* kinase-substrate relationships based on not only motif sequences but also various contextual factors [20]–[22]. The site-specific upstream kinases of regulated phosphorylation events identified by NetworKIN (Table S2) were further arranged into four subgroups according to their target motif sequences (Table S3 and Figure 3A). In Ras-transformed HBECs, kinases targeting proline-directed phosphosites represented the major subset in the phosphorylation network. A similar pattern was observed in AD cells (A549 and H322), but not in LCC H1299 cells (Figure 3A). Through a homogeneity test, the AD A549 and H322 cells appeared to share an undistinguishable pattern of upstream kinomes (p = 0.716). In contrast, the LCC H1299 cells displayed a more unique pattern, significantly different from that of the AD cells (p<0.0001), in which subsets of basophilic kinases and proline-directed kinases played equally important roles in the upstream kinomes (Figure 3A).

Supporting the above observations, both the LC-MS/MS and IDEAL-Q quantitative results and Western blot analysis showed increased phosphorylation and activation of proline-directed MAPK1 in Ras-transformed HBECs and AD cells, but not in LCC cells (Figure S2 and Figure 3B). Furthermore, the phosphorylation of PKN2, a basophilic kinase of the PKC family [23], was shown by LC-MS/MS analysis to be downregulated in AD cells, but not in LCC cells (Table S1, rows 427 and 428). Other basophilic kinases such as AKT [21], [24] have been reported to be upregulated in H1299 cells relative to levels in several AD cell lines, including A549 [25]. These data suggest that different subsets of kinomes are differentially involved in the pathogenic development of lung AD and LCC.

### Identification of phosphorylation targets of oncogenic Ras signaling in HBECs and NSCLC cells

By quantitative proteomics, we identified 52 proteins that were differentially phosphorylated at 77 sites in HBECs upon Ras-transformation (Table S4). Many of these proteins are known as downstream targets of MEK- or ERK-mediated signaling and include MAPK1 (ERK2) (at T185 and Y187), MAPK3 (Y204), cortactin (T364), FAM129B (S667), LMNA, MAP4, PAK4, and SRRM2 [26], [27]. Most importantly, many others were newly identified as potential targets of the Ras signaling pathway (Table S4). Western blot analyses were performed using site-specific antibody available to recognize the phosphorylated motif of lamin serine 392. In agreement with the mass spectrometric data, western blot showed upregulated phosphorylation of lamin at serine 392 in 3KTR relative to 3KT cells (Figure 3C), which is in agreement with the mass spectrometric data (Table 1). Attempts were also made to examine the phosphorylation status of cortactin by immunoprecipitating cortactin in cell lysates followed by immunobloting using antibody recognizing phosphorylated Ser/Thr residues. Although we can not pinpoint the phosphorylation of cortactin at threonine 364 residue, our data showed the upregulation of cortactin phosphorylation in 3KTR cells relative to 3KT (Figure 3C) (Table 1). Further, we performed the KRAS-knockdown experiments using Lentivirus-based approach to characterize the lamin pS392 status in three non-small cell lung cancer cells (A549, H322 and H1299) cultured in serum-free condition (Figure 3D). Introduction of two independent KRAS-shRNAs led to a significant decrease in the expression of KRAS protein in comparison to that of the control shRNA targeting at *luciferase* mRNA. In addition, knockdown of KRAS resulted in downregulation of lamin pS392 in adenocarcinoma A549 and H322 cells but not in large cell carcinoma H1299 cells. These results are in agreement with the mass spectrometry data, where we observed the upregulated lamin pS392 in A549 and H322 and but not in H1299 (Table 1).

**Table 1:** Identification of oncogenic Ras-regulated phosphorylation events that are concurrently regulated in adenocarcinoma A549 and H322 cells, but not in large cell carcinoma H1299 cells.

Gene symbol	Protein name	Swiss-Prot ID	Peptide sequence	Site	Log2 Ratio				
					3KTR/ 3KT	A549/ 3KT	H322/ 3KT	H1299/ 3KT
Ras stimulated phosphorylation events									
BAT3	Large proline-rich protein BAT3	P46379	APPQTHLPSGASSGTGSASA THGGG**pS**PPGTR	S113	1.718	1.680	2.423	0.806
			APPQTHLPSGASSGTG**pS**ASA THGGGSPPGTR	S104	1.717	1.664	2.400	0.776
			APPQTHLPSGASSGTGSASA **pT**HGGGSPPGTR	T108	1.715	1.679	2.420	0.803
BCLAF1	Bcl-2-associated transcription factor 1	Q9NYF8	SSATSGDIWPGLSAYDN**pS**PR	S222	1.104	1.612	1.176	0.688
			SSATSGDIWPGL**pS**AYDNSPR	S217	1.098	1.612	1.176	0.688
COBRA1	Negative elongation factor B	Q8WX92	KPSPAQAAE**pT**PALELPLPSV PAPAPL	T564	1.593	1.540	1.829	0.514
			KP**pS**PAQAAETPALELPLPSV PAPAPL	S557	1.589	1.531	1.827	0.477
CTTN	Cortactin	Q8N707	TQ**pT**PPVSPAPQPTEER	T364	1.065	1.052	1.236	−1.680
FAM129B	Niban-like protein	Q96TA1	AAPEAS**pS**PPASPLQHLLPGK	S679	2.107	1.752	1.556	−9999
LMNA	Lamin-A/C	P02545	LSP**pS**PTSQR	S392	1.737	1.102	1.475	0.977
			L**pS**PSPTSQR	S390	1.193	1.098	1.084	0.975
MAPK1	MAP Kinase, ERK2	P28482	VADPDHDHTGFLTE**pY**VATR	Y187	1.946	1.024	1.599	−1.308
MICAL1	Calponin and LIM domain containing 1	Q8TDZ2	LS**pS**PERQR	S829	1.737	1.108	1.220	0.972
PPP1R13L	RelA-associated inhibitor	Q8WUF5	SESAPTLHPYSPL**pS**PK	S113	1.218	1.235	1.688	0.532
SRRM2	Serine/arginine repetitive matrix protein 2	Q9UQ35	SR**pT**PPSAPSQSR	T2409	1.361	1.838	1.460	0.623
Ras repressed phosphorylation events									
ABI1	Abl interactor 1	Q8IZP0	LGSQH**pS**PGR	S225	−1.184	−1.181	−1.932	−0.669
API5	Apoptosis inhibitor 5	Q9BZZ5	RASEDTTSG**pS**PPKK	S464	−1.704	−3.756	−3.442	0.429
PALLD	Palladin	Q8WX93	**pS**PSGHPHVR	S880	−1.017	−1.676	−1.510	−0.586
TMEM40	Transmembrane protein 40	Q8WWA1	RG**pS**DPASGEVEASQLR	S153	−1.293	−5.644	−2.08	−9999

The regulated phosphorylation events are shown in Log_2_ scale. The relative phosphorylation levels with a 2-fold increase or decrease are indicated by bold font. The protein symbol, full name, and Swiss-Prot database protein accession number corresponding to each phosphopeptide, along with the phosphorylated residue location, are shown. “-9999” indicates peptides found in 3KT, but not in H1299.

Examination of the 52 Ras downstream phosphoproteins with the Panther Classification System revealed that most were involved in cell structure and motility, nucleic acid metabolism, cell cycle, signal transduction, or apoptosis according to their molecular function (Figure S3A) and biological process (Figure S3B). As Ras activation is a frequent event in lung adenocarcinoma [1], [3], we next looked at the status of Ras-regulated phosphorylation events in the three NSCLC cell lines. In agreement with the observation of enhanced MAPK activation in AD cells, but not in LCC cells, about 50% of the Ras-upregulated proline-directed sites (n = 31) were upregulated in AD cells (A549 and H322), whereas only 6% were upregulated in LCC cells (H1299) (Figure 3E and Table S4). Among the 52 Ras-regulated proteins, 14 were concurrently found to be regulated in AD A549 and H322 cells, but not in LCC H1299 cells (Table 1). These data further implicated the involvement of Ras-mediated pathways in the pathogenic development of lung AD.

### Identification of Ras-regulated pathways in HBECs and their involvement in lung AD, but not LCC

**Figure 4:**
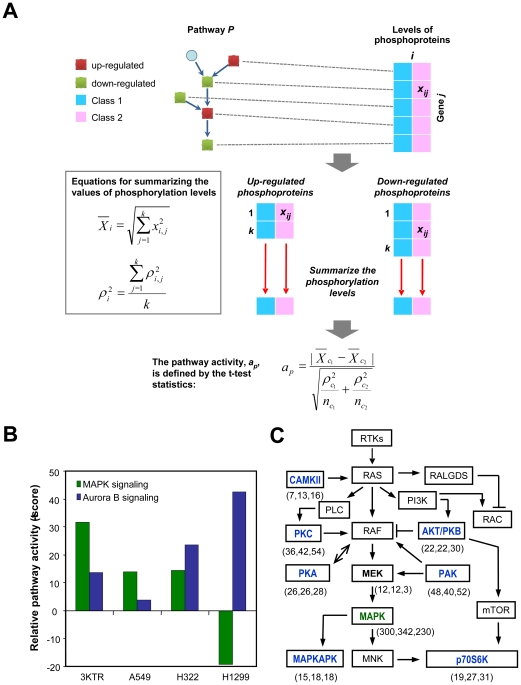
Inferring pathway activity in Ras-transformed HBECs and NSCLC cells. (A) The levels of regulated phosphoprotein signatures obtained from quantitative proteomics analysis were subjected to pathway activity analysis. Equations for summarizing the levels of phosphorylated proteins and for analyzing pathway activity are shown. *x_i,j_* and *ρ_i,j_* are the mean and standard deviation, respectively, of the value of the phosphoprotein *j* in class *i,* and *k* is the number of proteins in a given pathway. *a_p_* is the activity score for pathway *p* between classes *c_1_* and *c_2_*, and 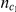 and 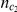 are the numbers of replicate experiments for the respective classes. In our case, 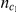 and 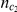
are each 3. (B) Inferred activities of MAPK and Aurora B signaling pathways in Ras-transformed HBECs and NSCLC cells in comparison with HBEC-3KT are shown as relative pathway activity scores (t-scores). (C) The involvement of the Ras/Raf/MEK/MAPK signaling pathway and its cross-talk with basophilic kinases in NSCLC cells. Kinases identified as being upstream of the regulated phosphosites by NetworKIN analysis in this study are highlighted in bold font. Kinases targeting proline-directed and basophilic S/T sites are labeled in green and blue letters, respectively. The frequencies of the individual kinases identified in A549, H322, and H1299 cells in comparison with HBEC-3KT are shown in parentheses.

Our data showed that Ras-regulated phosphorylation events are conserved in AD cells, and thus we further inferred the activation status of pathways, including MAPK signaling, in these cells by integrating the levels of the regulated phosphorylation events into individual signaling pathways according to the NCI-Nature Pathway Interaction Database (PID). For each signaling pathway, an activity level was summarized from the phosphorylation levels of the subset of proteins in the pathway, following the method reported by Lee et al. with modifications [15]. Figure 4A shows a schematic representation and equations for the pathway activity analysis. Pathways were included in the activity analysis only when they consisted of five or more identified phosphoproteins. The pathway activity signatures identified in each pair of cell lines are listed in Table S5. We observed 23 upregulated and two downregulated pathways downstream of oncogenic Ras in HBECs. The upregulated pathways included growth factor-mediated signaling pathways from HGF, FGF, and VEGF. The computation of pathway activities revealed the upregulation of proline-directed kinase MAPK signaling in 3KTR and AD cells (A549 and H322), but not in LCC H1299 cells (Figure 4B). In contrast, higher activity of the basophilic kinase Aurora B-mediated signaling pathway was observed in H1299 cells compared with A549 and H322 cells (Figure 4B).

In the subgroup of upstream kinases targeting proline-directed sites, the MAPK family represented the major upstream species, followed by CDK and GSK (Table S3). The subgroup of kinases targeting basophilic phosphosites included PAK, PKC, DMPK, CLK, Pim, PKA, and PKB. Unlike the pattern of kinases upstream of the regulated phosphorylation events in the Ras-transformed HBECs and AD cells, a higher percentage of basophilic kinases was observed in the LCC H1299 cells (Figure 3A). Interestingly, many of the basophilic kinases that were over-activated in H1299 cells were directly or indirectly linked to the Ras/Raf/MAPK pathway (Figure 4C), suggesting that the interplay between the Ras/Raf/MAPK signaling axis and basophilic kinases may dominate the pathogenic development of NSCLC.

### Identification and functional significance of Ras signaling-regulated phosphoproteins and pathways

We next established protein interaction networks to identify the physical and functional interactions among the proteins that are phosphorylated by Ras-mediated signaling. The proteins (n = 584) harboring the phosphopeptides (n = 1288) that were subjected to comparative proteomics analysis between normal HBECs and Ras-transformed HBECs were further analyzed using the Search Tool for the Retrieval of Interacting Genes/Proteins (STRING) database of physical and functional interactions. This yielded a phosphoprotein interaction network consisting of 1279 known plus predicted protein interactions among 401 proteins (Figure S4). In this protein interaction network, we identified 45 proteins that were previously described as direct targets of MAP kinase [28]. Most importantly, 40 of the 52 Ras-regulated phosphoproteins identified in this study were found in the protein interaction network (Figure S4). The establishment of a protein-protein interactome suggests that downstream phosphorylation targets of oncogenic Ras/MAPK-mediated signaling are closely associated in their molecular function.

In a large-scale proteomics study, identifying kinase substrates is difficult owing to the unavailability of antibodies. The NetworKIN algorithm provides a highly accurate alternative approach for the prediction of site-specific upstream kinases [20]-[22]. Among the 40 Ras-regulated proteins found in the protein interaction network, 14 were identified by NetworKIN analysis as novel substrates for MAP kinases (Figure S4 and Table S4). Of the newly identified MAPK target sites, COBRA1 (S557), LMNA (S392), LMO7 (S1259), and WRNIP1 (S153) represent bona fide targets of MAPKs with high substrate specificity (Table S2). In the case of LMNA, our study specifically revealed its enzyme activation site (S392) as the target of oncogenic Ras-mediated MAP kinase signaling. To further exclude the possibility that the regulated phosphorylation resulted from differential expression of these proteins, we analyzed the expression status of the corresponding genes in a microarray dataset (NCBI's GEO database, Reference GSE11969) related to clinical samples, where normal lung (n = 5) and adenocarcinoma samples with (n = 12) and without (n = 84) KRAS mutations were compared. These genes were expressed at comparable levels in lung carcinomas irrespective of the status of KRAS mutations (Table S6). These data suggest that oncogenic Ras-mediated MAPK signaling is involved in the elevated phosphorylation of these bona fide target proteins.

Finally, the identified Ras-regulated pathways were clustered based on their biological processes and function similarities. Figure S5 shows the hierarchical clustering analysis of the 23 signaling pathways regulated by oncogenic Ras. A heat map was generated to show the functional associations among the pathways according to functional similarity scores ranging from 1.0 to 0.06. We identified four clusters of tightly associated pathways with high similarity (≥0.5) among the Ras-activated signaling pathways (Figure S5). The clusters represented the regulation of RNA processing, apoptosis, cell growth, and gene expression, respectively. Moreover, pathways associated with MAP kinase signaling were identified with a half-maximum functional similarity score (≥0.5) as a threshold. In addition to the HGF-mediated signaling pathway (functional similarity score of 0.505), others, including the BCR (0.548), keratinocyte receptor (0.52), agrin (0.493), and Fc-epsilon receptor (0.492) pathways, were inferred in this study as novel pathways associated with Ras/MAP kinase signaling (Table S7). The results of the pathways associated with oncogenic Ras signaling correspond well to previously described molecular functions of Ras signaling [3], [4], [29]. In contrast, the downregulated pathways observed in Ras-transformed HBECs did not show any association.

## Discussion

Using mass spectrometry-based comparative phosphoproteomics, we established the phosphorylation profiles of five lung epithelial cell lines. Comparison of HBECs upon Ras-transformation revealed that proline-directed kinases, which include the MAPK family, are the major mediators of Ras downstream signaling. The comparisons of the upstream kinases in three NSCLC cell lines demonstrated that proline-directed and basophilic kinases play important roles in the regulation of phosphorylation events in AD and LCC cells. Further, we inferred Ras-regulated pathways and their functions by introducing an algorithm, which supports the stimulation of Ras/MAPK signaling in lung adenocarcinoma but not in LCC. Our data demonstrate Ras-regulated phosphorylation events and pathways, as well as the upstream kinomes, and thereby provide new insights into Ras-mediated signaling networks in lung adenocarcinoma.

Quantitative comparisons of phosphopeptides across five cell lines were performed using the IDEAL-Q system. For any cell line, >95% of the phosphopeptides were quantified in the other four cell lines, which is higher than previously reported, where only 63% of the identified peptides of the five samples were shown using label-free quantitation 8]. The improved sensitivity of label-free quantitation shown here is adaptable to any system analyzing multiple samples. In our study, the number of regulated phosphorylation events was much lower in the comparison of 3KT and 3KTR cells (Figure 2A). This is consistent with the nature of their isogenic background and further demonstrates the usefulness of the immortalized HBECs in studying the phosphoproteomic profiles of oncogenic signaling 6]. A majority of the Ras-regulated phosphosites identified in HBECs consisted of proline-directed sequences. More than 50% of these proline-directed phosphosites were upregulated in adenocarcinoma A549 (harboring the *KRAS^G12D^* mutation) and H322 (*KRAS* amplification) cells, but not in LCC H1299 (*NRAS^Q61K^*) cells (Figure 3E and Table 1), in agreement with previous reports that oncogenic KRAS, but not oncogenic NRAS, is effective in stimulating downstream MAPK signaling 30,31]. In addition, the upregulation and downregulation of MAPK phosphorylation were observed in AD and LCC cells, respectively, by LC-MS/MS analysis (Figure S2) and immunoblotting (Figure 3B). In the NetworKIN analysis, we showed that the involvement of basal kinases including CAMKII, PKA, AKT/PKB, PKC, PAK, and p70S6K was significantly increased in H1299 cells relative to A549 and H322 cells (Figure 3A). The increase in basophilic kinases may lead to the attenuation of Ras-mediated MAPK signaling output in H1299 cells (Figure 4C). Our data suggested that MEK/MAPK kinases in adenocarcinoma cells where as downstream kinases of PI3K and PLC including AKT/PKB, p70S6K and PKC in large cell carcinoma cells play key roles in regulating the protein phosphorylation events (Figure 4C).

Seventy-seven phosphorylation events in 52 proteins were significantly altered by oncogenic Ras signaling in HBECs. Among these proteins, 14 were identified as novel targets of MAPK family kinases by NetworKIN analysis (Figure S4). These proteins are involved in diverse biological processes and molecular functions, including cytoskeletal organization (ABI1, KRT5, LMO7, and MICAL1); nucleoskeletal organization (LMNA); small GTPase-mediated signal transduction (SIPA1L1); ubiquitin-involved signal transduction (UBAP2L); regulation of ubiquitination (STUB1), acetylation processes (BAT3), DNA replication (MCM2), and chromatin de-condensation and DNA synthesis (WRNIP1); and negative regulation of transcription elongation (COBRA1). NetworKIN analysis specifically revealed COBRA1 (S557), LMNA (S392), LMO7 (S1259), and WRNIP1 (S153) as the site-specific phosphorylation targets of MAPK with relatively high probability (Table S2). Among these, COBRA1 and LMNA were upregulated in 3KTR, A549, and H322 cells, but not in H1299 cells (Table S4). However, the upregulation of LMO7 was observed in 3KTR, A549, H322, and H1299 cells. The regulated phosphopeptide of LMO7 contains the sequence motif R-X-X-[pS]-P, suggesting that an unknown basophilic kinase may be involved in its regulation (Table S4). WRNIP1 was downregulated in 3KTR cells, but upregulated in H1299 cells (Table S4). In this study, residue S392 of LMNA, which is located at the enzyme active site, was identified as a novel site-specific target of oncogenic Ras-mediated MAPK signaling. The downregulation of WRNIP1 phosphorylation levels suggests cross-talk between oncogenic Ras signaling and other pathways in controlling biological processes through negative regulation of protein phosphorylation.

Understanding altered signaling pathways that regulate cellular processes is a basis for drug discovery 32]. The identification of deregulated pathways, rather than genes, as biomarkers would give precise information of clinical value. Several studies have demonstrated pathway signatures based on the transcriptome, transcription factors, or proteome 33,34]. Given that phosphorylation plays a major role in regulating signaling pathways that dominate the pathogenic development of malignancies, we demonstrated a new strategy to identify pathways altered at the phosphorylation level. We inferred pathway activities, which revealed the activation of MAPK signaling in Ras-transformed HBECs. We further showed that MAPK signaling was upregulated in A549 and H322 cells, but not in H1299 cells (Figure 4B). These results are consistent with previous reports of mutations and amplifications of KRAS leading to activation of downstream signaling in lung adenocarcinomas 3]. Clustering the upregulated pathways identified in Ras-transformed HBECs revealed that molecular functions such as RNA processing, apoptosis, cell growth, and gene expression are regulated by oncogenic Ras signaling. Pathway correlation was performed for MAPK signaling, and correlated pathways, including the agrin pathway, BCR pathway, Fc-epsilon receptor pathway, HGFR signaling, and keratinocyte pathway, were identified using a correlation threshold of 0.5 (Figure S5). These findings unravel known and previously unknown pathways and biological processes that are closely associated with oncogenic Ras-mediated signaling.

## Supporting Information

Figure S1 **Identification of phosphopeptides, coefficient of variation (CV) of phosphopeptide abundances, and normalization process.** (A) Whole cell lysates were prepared from each cell line, mixed with β-casein, digested by trypsin, and phosphopeptides were enriched by IMAC. The enriched phosphopeptides from each cell line was analyzed by LC-MS/MS for three times. % of identified phosphopeptides in triplicate runs is shown. (B) % of CV of quantifiable phosphopeptide abundances in three runs are shown for five cell lines. (C) The observed abundance of phosphopeptide (^49^FQpSEEQQQTEDELQDK^63^) derived from β-casein in three LC-MS/MS runs is shown for five cell lines. (D) Normalization process is shown for a case comparison, 3KTR vs 3KT. Phosphopeptide ratios (in Log_2_ scale) were obtained in the comparison of 3KTR and 3KT cells after normalized against β-casein, internal standard intensity. The phosphopeptide ratios are shown on X-axis and the number of phosphopeptides is shown on Y-axis. (E) The ratios normalized with internal standard were then log-centered.
(TIF)

Figure S2 **MAPK phosphorylation observed by LC-MS/MS.** Quantitative assessment of the relative phosphorylation status of ERK at T185 and Y187 residues in 3KTR, A549, H322, and H1299 cells in comparison to 3KT cells by LC-MS/MS and IDEAL-Q.
(TIF)

Figure S3 (A) Classification of Ras-regulated phosphoproteins according to their molecular function. (B) Classification of Ras-regulated phosphoproteins according to their biological processes. The classification was made using the database, Panther Classification System, available on http://www.pantherdb.org/.
(TIF)

Figure S4 **Functional interaction network of the phosphoproteins in Ras-transformed HBECs.** Phosphoproteins identified in Ras-transformed HBECs were subjected to STRING database with medium confidence (≥0.4) applied. The upregulated and downregulated phosphoproteins involved in oncogenic Ras signaling are marked in pink and green boxes, respectively. The known and novel substrates of MAP kinases identified by NetworKIN analysis are marked in boxes circumscribed by blue and red, respectively.
(TIF)

Figure S5 **Clustering of Ras-regulated signaling pathways.** Hierarchical agglomerative average-linkage clustering analysis of oncogenic Ras-regulated pathways identified in HBECs was performed based on their gene ontology functional similarity. Pearson's correlation coefficient was derived as the distance matrix and visualized by Generalized Association Plots (GAP). The color bar indicates the increasing association among the pathways with increasing color intensity from blue to red. The rows and columns show the pathways in similar order, and the association between two identical pathways is shown as a red line throughout the map. Four pathway clusters were identified, depicted in thick red lines on the right side, using a correlation threshold of 0.5.
(TIF)

Table S1 **The list of quantifiable phosphopeptides across five cell lines are shown here.** Upon comparisons, phosphopeptides showing up- and down-regulated levels are highlighted in red and green colors, respectively. Data corresponding to each peptide identified by mass spectrometry and Mascot search, including peptide sequence, m/z value, charge, modification site, international protein index (IPI) accession number, average abundance and standard deviation, are shown. Few peptides were not quantified by IDEAL-Q and such peptides are indicated as NaN, -9999, and 9999. In the case of 3KTR vs 3KT, if the peptide was not identified in both cell lines or identified in 3KTR only or identified in 3KT only then they are indicated as NaN, 9999 and -9999, respectively.
(XLS)

Table S2 **The list of site specific upstream kinases of regulated phosphorylation events predicted by NetworKIN.** The regulated phosphorylated peptides identified in the cases of 3KTR or A549 or H322 or H1299 vs 3KT were subjected to NetworKIN analysis. List of site specific upstream kinases predicted by NetworKIN analysis are shown in this table. For each regulated peptide, Swiss-Prot accession number of the protein, phosphorylation site, upstream kinase family, kinase ID, kinase symbol, NetPhorest posterior probability, String score, NetworKIN score of the kinase, are shown.
(XLS)

Table S3 **Frequency of upstream kinases of regulated phosphorylation events identified in 3KTR, A549, H322 and H1299 cells.** The upstream kinases of regulated phosphorylation events identified in individual pairs of cell lines were predicted by NetworKIN analysis. According to their target sequences, the upstream kinases were grouped into subsets of proline-directed, basophilic, acidophilic, and other kinases. The kinases targeting other than proline-directed, basophilic and acidophilic S/T sites are shown. The frequencies of subsets of kinases are listed.
(XLS)

Table S4 **The oncogenic Ras-regulated phosphorylation events were identified in 3KTR and their status is shown in NSCLC cells.** The regulated phosphorylation events in HBECs upon oncogenic Ras-transformation (3KTR vs 3KT) are shown in Log_2_ scale. The relative levels of these phosphorylation events in NSCLC cells (A549 or H322 or H1299 vs 3KT) in relation to 3KT are shown. The regulated phosphosites were listed as proline-directed, basophilic, acidophilic and others. The relative phosphorylation levels with 2-fold increase or decrease are indicated by bold font. Few peptides were not quantified by IDEAL-Q and such peptides were indicated as NaN, -9999, and 9999. In the case of 3KTR vs 3KT, if the peptide was not identified in both cell lines or identified in 3KTR only or identified in 3KT only then they were indicated as NaN, 9999 and -9999, respectively. The protein symbol, full name, and Swiss-Port database protein accession number corresponding to each phosphopeptide, along with phosphorylated residue location, and NetworKIN assessment of upstream kinases are shown. In the table, (A) indicates peptides listed here only when they regulated in 3KTR in relation to 3KT with exception for MAPK1 T185, MAPK3 Y204 and MAPK14 Y182 peptides. (B) indicates MEK1/2 kinases were not covered by NetworKIN. (C) indicates MAP kinases are listed for the regulated sites according to the NetworKIN specificity. (I) indicates isoform.
(XLS)

Table S5 **The list of pathway activities inferred in Ras-transformed HBECs and NSCLCs.** The expression values of up- and down-regulated phosphorylation events were integrated in Pathway Interaction Database to examine the up- and down-regulated pathway activity signatures respectively. Pathway activities observed in four comparisons (3KTR or A549 or H322 or H1299 vs 3KT) were listed. For each identified regulated pathway, pathway ID, number of phosphoproteins identified, and number of regulated phosphoproteins identified, pathway activity score, are shown. The pathways consisting more than five identified phosphoproteins were considered for pathway activity analysis.
(XLS)

Table S6 **The expression levels of bonafide MAPK targets and MAP kinases in lung adenocarcinomas with and without KRAS mutations.** The relative expression levels of bonafied MAPK targets and MAP kinases are shown by comparing lung adeoncarcinomas with (n = 12) and without (n = 84) KRAS mutations to normal tissues (n = 5). This data is derived from Agilent Homo sapiens 21.6K custom array, the data values were corrected for background and normalized, and the data was deposited on NCBI's GEO oligo microarray database (Data series GSE11969). Upon comparisons, proteins showing up- and down-regulated levels are highlighted in red and green colors, respectively.
(XLS)

Table S7 **Identification of pathways associated with Ras-signaling by pathway clustering.** Upregulated pathways (n = 23) identified in Ras-transformed HBECs in relation to normal HBECs (3KTR vs 3KT) were clustered based on their gene ontology functional similarities. Correlation values observed among the upregulated pathways in Ras-transformed HBECs are shown. The maximum correlation score was one, which is seen between the same pathways. The rows and columns show the pathways in similar order therefore association between two identical pathways shown a red line throughout the map (see Figure S5).
(XLS)

Methods S1 **Supplementary Methods.**
(DOC)
