# Supplementary figures and images for: Phosphoproteomics Identifies Oncogenic Ras Signaling Targets and Their Involvement in Lung Adenocarcinomas
Source: PLoS One. 2011 May 26;6(5):e20199. doi: 10.1371/journal.pone.0020199 (PMC3102680; doi:10.1371/journal.pone.0020199)

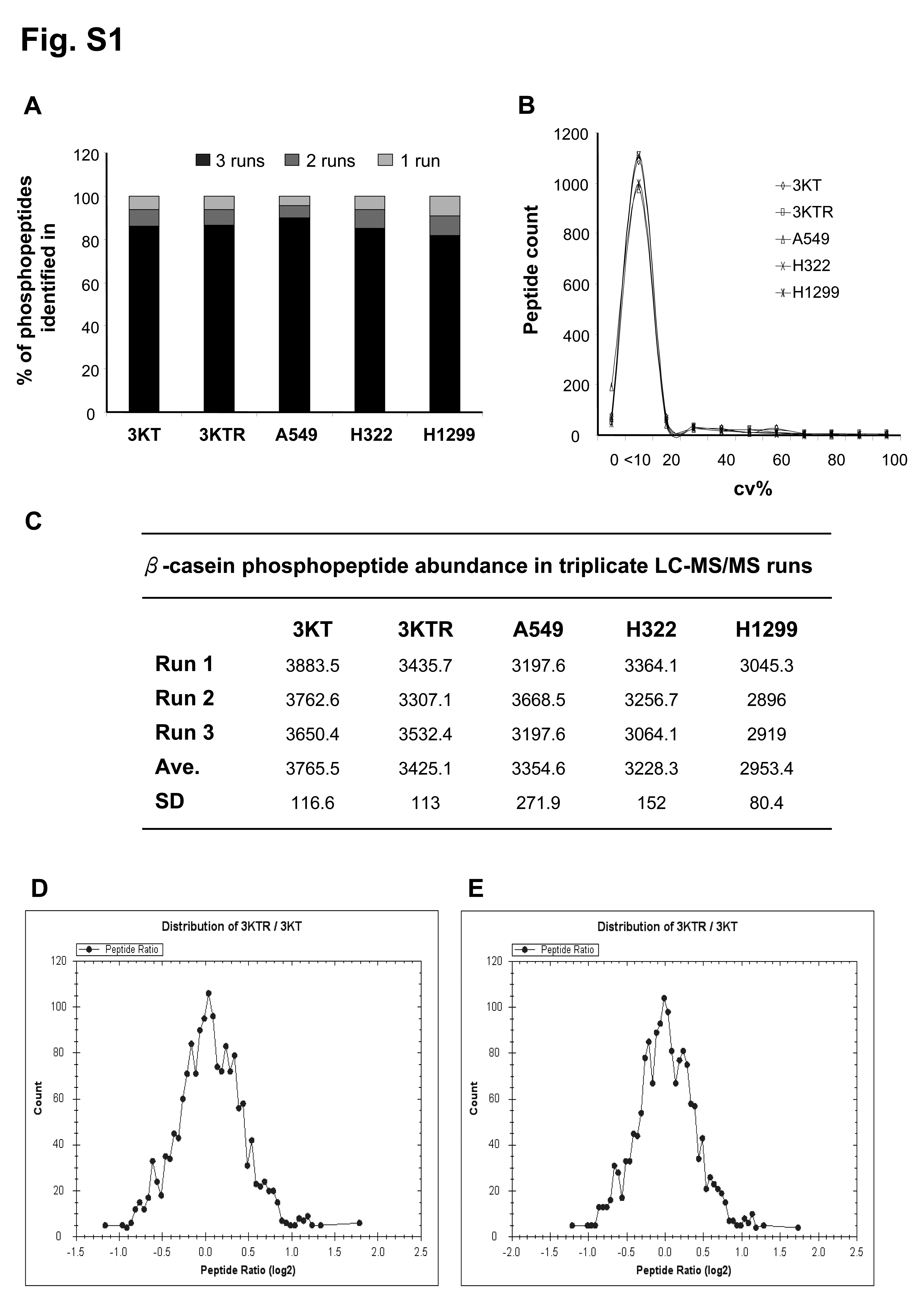

Supplement: Figure S1 — Identification of phosphopeptides, coefficient of variation (CV) of phosphopeptide abundances, and normalization process. (A) Whole cell lysates were prepared from each cell line, mixed with β-casein, digested by trypsin, and phosphopeptides were enriched by IMAC. The enriched phosphopeptides from each cell line was analyzed by LC-MS/MS for three times. % of identified phosphopeptides in triplicate runs is shown. (B) % of CV of quantifiable phosphopeptide abundances in three runs are shown for five cell lines. (C) The observed abundance of phosphopeptide (49FQpSEEQQQTEDELQDK63) derived from β-casein in three LC-MS/MS runs is shown for five cell lines. (D) Normalization process is shown for a case comparison, 3KTR vs 3KT. Phosphopeptide ratios (in Log2 scale) were obtained in the comparison of 3KTR and 3KT cells after normalized against β-casein, internal standard intensity. The phosphopeptide ratios are shown on X-axis and the number of phosphopeptides is shown on Y-axis. (E) The ratios normalized with internal standard were then log-centered. (TIF) [file pone.0020199.s001.tif]

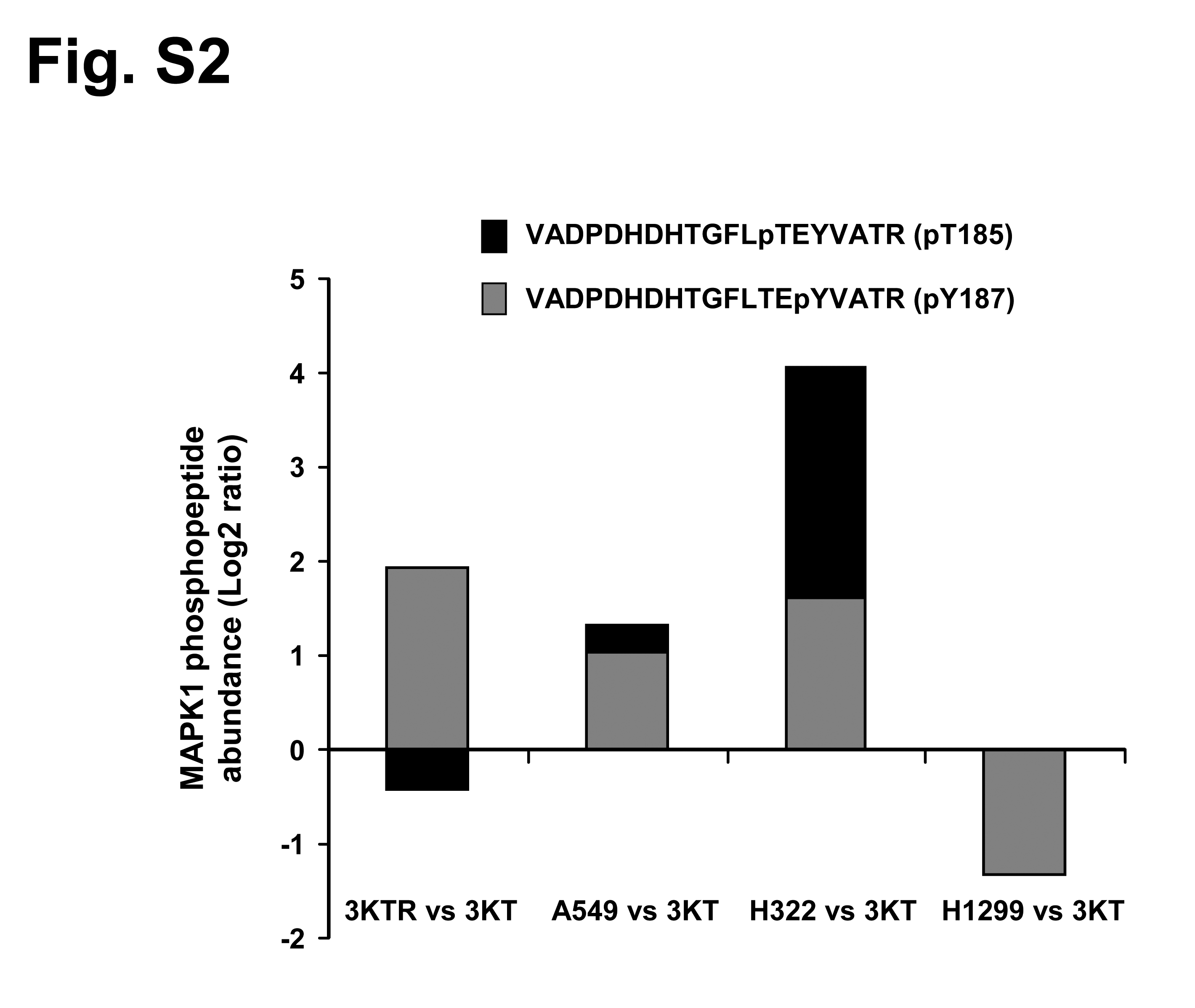

Supplement: Figure S2 — MAPK phosphorylation observed by LC-MS/MS. Quantitative assessment of the relative phosphorylation status of ERK at T185 and Y187 residues in 3KTR, A549, H322, and H1299 cells in comparison to 3KT cells by LC-MS/MS and IDEAL-Q. (TIF) [file pone.0020199.s002.tif]

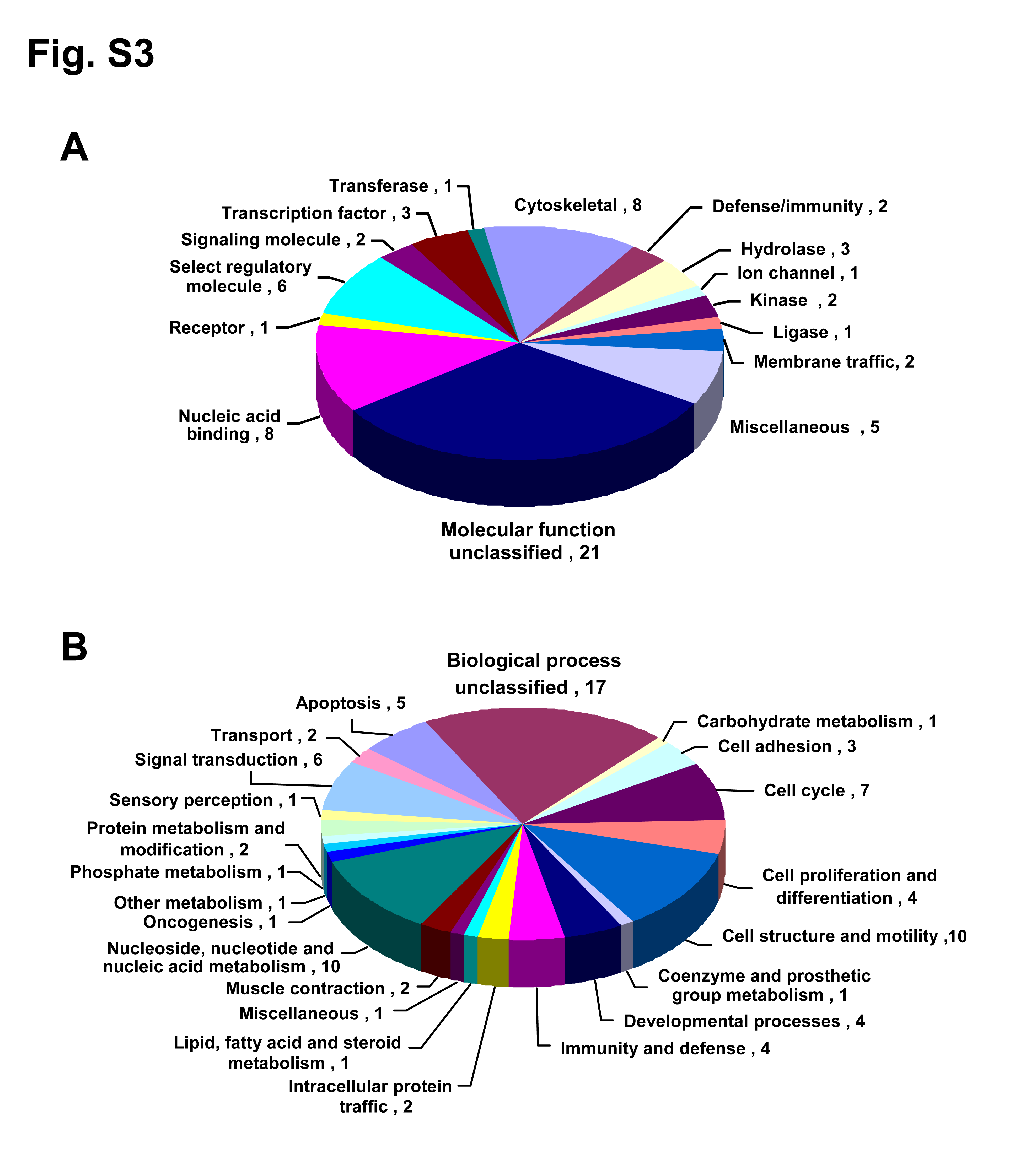

Supplement: Figure S3 — (A) Classification of Ras-regulated phosphoproteins according to their molecular function. (B) Classification of Ras-regulated phosphoproteins according to their biological processes. The classification was made using the database, Panther Classification System, available on http://www.pantherdb.org/. (TIF) [file pone.0020199.s003.tif]

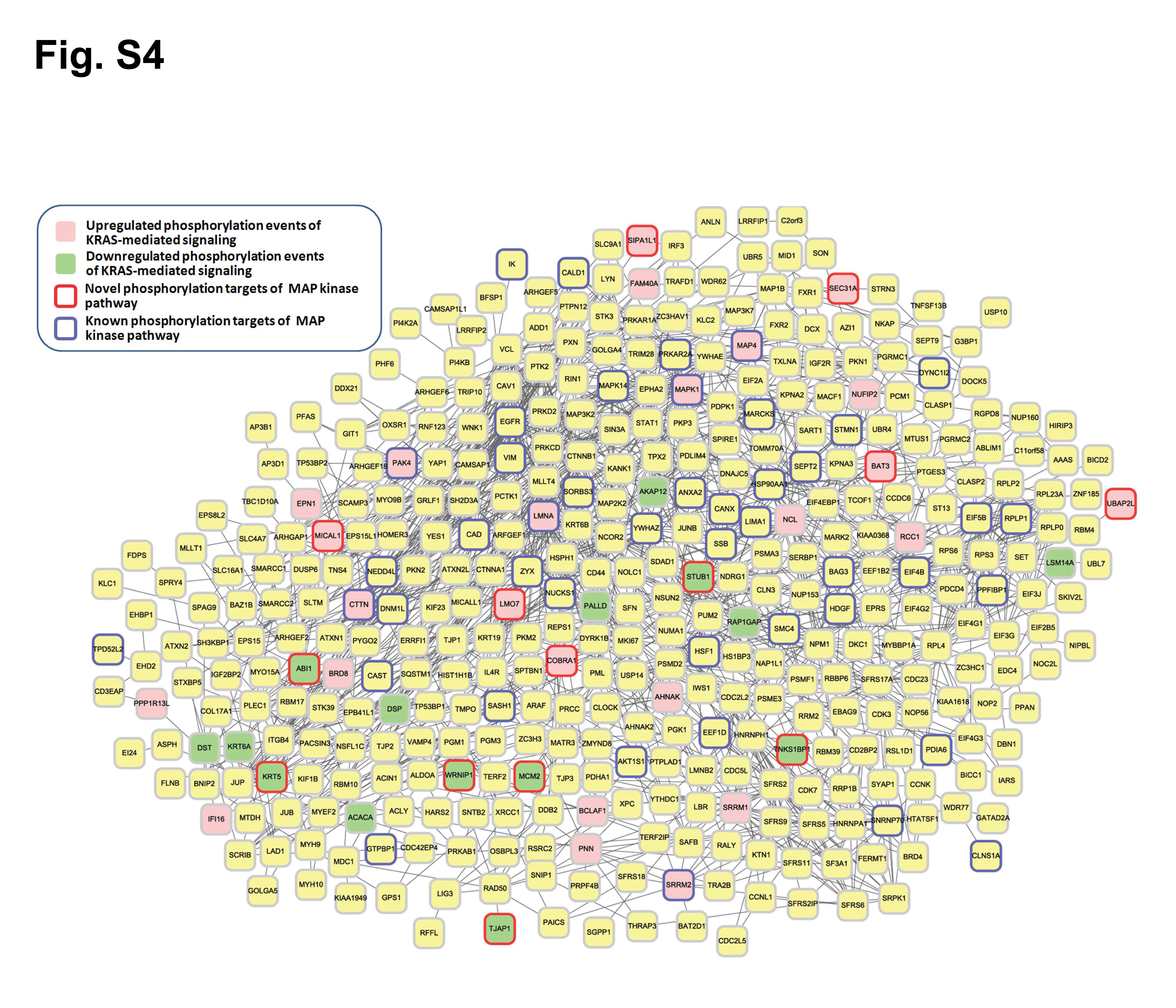

Supplement: Figure S4 — Functional interaction network of the phosphoproteins in Ras-transformed HBECs. Phosphoproteins identified in Ras-transformed HBECs were subjected to STRING database with medium confidence (≥0.4) applied. The upregulated and downregulated phosphoproteins involved in oncogenic Ras signaling are marked in pink and green boxes, respectively. The known and novel substrates of MAP kinases identified by NetworKIN analysis are marked in boxes circumscribed by blue and red, respectively. (TIF) [file pone.0020199.s004.tif]

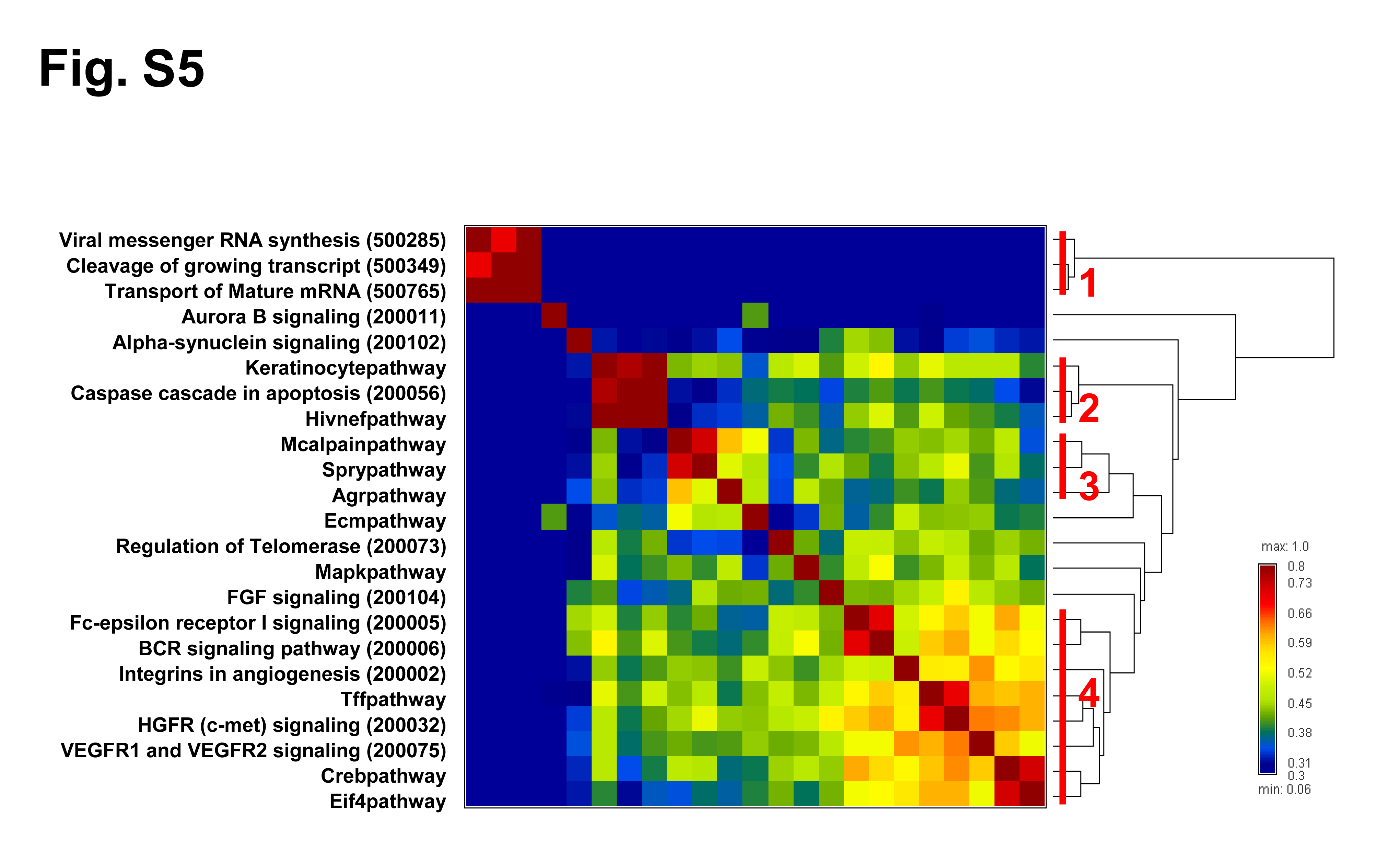

Supplement: Figure S5 — Clustering of Ras-regulated signaling pathways. Hierarchical agglomerative average-linkage clustering analysis of oncogenic Ras-regulated pathways identified in HBECs was performed based on their gene ontology functional similarity. Pearson's correlation coefficient was derived as the distance matrix and visualized by Generalized Association Plots (GAP). The color bar indicates the increasing association among the pathways with increasing color intensity from blue to red. The rows and columns show the pathways in similar order, and the association between two identical pathways is shown as a red line throughout the map. Four pathway clusters were identified, depicted in thick red lines on the right side, using a correlation threshold of 0.5. (TIF) [file pone.0020199.s005.tif]
