# Supplementary material for: Phosphoproteomics Identifies Oncogenic Ras Signaling Targets and Their Involvement in Lung Adenocarcinomas
Source: PLoS One. 2011 May 26;6(5):e20199. doi: 10.1371/journal.pone.0020199 (PMC3102680; doi:10.1371/journal.pone.0020199)
Supplement: Methods S1 — Supplementary Methods. (DOC) [file pone.0020199.s013.doc]

**Supplementary Information Text S1**

**Methods**

**Western blot analysis**

Immunoblotting was performed as described earlier [11]. Briefly, we used primary antibodies including rabbit polyclonal anti-phospho-ERK (T185, Y187; Cell Signaling Technology), rabbit polyclonal anti-ERK (Cell Signaling Technology), rabbit polyclonal anti-phospho-Lamin-A/C (S392; Abcam), mouse monoclonal-anti-Lamin-A/C (Santa Cruz Biotechnology), mouse monoclonal anti-Cortactin (p80/85), clone 4F11 (Millipore), mouse monoclonal anti-pS/T-P (MPM2; Upstate), mouse monoclonal anti-KRAS (Santa Cruz Biotechnology), mouse monoclonal anti-β-actin (Sigma). Primary antibodies were detected by using a goat anti-rabbit IgG secondary antibody conjugated with horse radish peroxidase (HRP) (Chemicon) or goat anti-mouse IgG-HRP from Pierce. Finally, the images were obtained from image reader LAS-3000 (Fujifilm).

**Tube-gel digestion and peptide extraction**

Total cell lysate from each cell line was mixed with bovine β-casein, and subjected to tryptic digestion. Tube-gel digestion was performed as described previously [12] with modifications. In brief, whole cell lysate was mixed with 40% acrylamide solution in an eppendorf tube. The solidified gel matrix was cut into small pieces and washed with 25 mM TEABC with 50% ACN for 30 min each time for 3 times to remove the detergents. After washing with 100% ACN for 2 min, the gel pieces were dried completely in SpeedVac. Two folds to the initial gel matrix volume of 25 mM TEABC buffer was added, and 0.5 µg of β-casein was added as an internal standard. Proteolytic digestion was performed overnight at 37°C using sequence grade modified trypsin (Promega) where the protein to trypsin ratio was 40:1. Peptides were extracted for 3 times using 5% formic acid and 50% ACN for 30 min each time.

**Phosphopeptides enrichment**

Phosphopeptides were purified using a revised one-step IMAC as described previously [13]. The Ni-NTA resin was washed with 0.1% acetic acid and packed into a 5-cm microcolumn (500 µm i.d. PEEK column, Upchurch Scientific/ Rheodyne, Oak Harbor, WA). The column was washed with condition buffer (6% acetic acid, pH 3.0) containing 50 mM EDTA and 1M NaCl for 15 min, then activated with condition buffer containing 0.2 M FeCl_3_ and equilibrated with the same buffer for 30 min. Peptide sample was dissolved in condition buffer at final pH as 3.0. After loading, the column was washed with condition buffer and ACN (3:1, v/v) for 15min and then with condition buffer for 20 min. Peptides were eluted by 0.2 M NH_4_H_2_PO_4_ (pH 4.4) for 15 min. The eluents were dried in SpeedVac and desalted by ZipTip prior to LC-MS/MS analysis.

**Mass spectrometry analysis**

Purified phosphopeptide samples were reconstituted in 4 μl buffer A (0.1% formic acid in H_2_O) and analyzed by LC-MS/MS (Waters Q-TOFTM Premier from Waters Corp, Milford, MA). Samples were injected into a 2 cm × 180 μm capillary trap column and separated by a 25 cm × 75 μm Waters1 ACQUITYTM 1.7 μm BEH C18 column using the nanoACQUITY Ultra Performance LCTM system (Waters Corp., Milford, MA). The column was maintained at 35^o^C and bound peptides were eluted with a linear gradient of 0–80% buffer B (buffer A, 0.1% FA in H_2_O; buffer B, 0.1% FA in ACN) for 120 minutes. MS was operated in ESI positive V mode with a resolving power of 10,000. The NanoLockSpray source was used for accurate mass measurement and the lock mass channel was sampled every 30 seconds. The mass spectrometer was calibrated with a synthetic human [Glu1]-Fibrinopeptide B solution (1 pmol/μL, from Sigma Aldrich) delivered through the NanoLockSpray source. Data was acquired via data directed analysis (DDA). The method included a full sequential MS scan (m/z 400-1600, 0.6 second) and 3 MS/MS (m/z 100-1990, 1.2 seconds per scan) on the three most intense ions present in the full scan mass spectrum.

**Peptide identification by Mascot searching**

LCQ-TOF MS/MS data were processed using Mascot Distiller version 2.0 (Matrix Science, London, UK; version 2.2.1). Mascot search options used in this study were ipi_HUMAN_3.29 database, digestion enzyme trypsin, fragment ion mass tolerance of 0.1 Da and a parent ion tolerance of 0.3 Da. Variable modification including oxidation of methionine and phosphorylation of serine, threonine and tyrosine were considered. Swisssprot_Metazoa_Animals database (version 54.2, 17,170 entries) was used for identification of β-casein. Peptides ions were filtered based on the p-value <0.05.

**Quantitative analysis by IDEAL-Q algorithm**

The quantitative analysis of phosphopeptide in the label-free experiments was performed by IDEAL-Q software [14]. The raw data files acquired from Waters Q-TOF^TM^ Premier were converted into files of mzXML format by the program massWolf, and the search results in MASCOT were exported in eXtensible Markup Language data (XML) format. After data conversion, the peptide identification results from each LC-MS/MS run were loaded and processed to establish a global peptide information list according to the criteria described in IDEAL-Q. First, prediction of elution time for the unidentified ions is performed based on the peptide entry list using linear regression in different LC-MS/MS runs followed by a fragmental correction function. Thus, the unidentified peptide ions due to low abundance or limitation of duty cycle of mass spectrometers can be detected and assigned. To ensure correct assignment, the detected peptide peaks were validated by the SCI validation using the following criteria: (a) signal-to-noise (S/N) ratio > 3, (b) correct charge state and (c) correct isotope pattern. To calculate relative peptide abundance, the tool uses area under curve of extracted ion chromatography (XIC), and the calculated XIC area is then normalized by the XIC area of internal standard.

**Pathway functional clustering**

Pathways were clustered based on their functional similarity. The functional similarity was computed for each pathway pair based on the Gene Ontology (GO) annotations of proteins in each pathway. We represent a pathway by a specific vector *p* as follows:

$$p_{j}=(w_{1,j},w_{2,j},...,w_{n,j})$$

where *w_i,j_* is the weighted value that the GO term *i* takes for pathway *j.* We only considered biological process ontology to construct vectors. The weighting values are derived from two parts: the local and global weights of a GO term. The local weight, *lw_i,j_*, is given by

$$lw_{i,j}=tf_{i,j}+\sum_{t\in children(i)} tf_{t,j}\times cf_{i,t}$$

where *tf_i,j_* is the number of proteins annotated by the GO term *i* in the pathway *j*, *children(i)* is the set of child nodes of term *i*, and *cf_i,t_* is the contribution function for terms *i* and *t*. The contribution function is based on the distance between terms on the GO graph and is formulated as

$$cf_{i,t}=\frac{dist_{i,root}}{dist_{i,t}+dist_{i,root}}$$

where *dist_i,t_* is the shortest path distance on the GO graph between the two terms *i* and *t*. The *root* corresponds to the root term. In our case, the root term is “biological process.” The global weight of term *i*, *gw_i_*, is calculated by

$$gw_{i}=\log\frac{N}{n_{i}}$$

where *N* is the total number of proteins in a given organism, and *n_i_* is the number of proteins annotated by term *i*. Finally, because of the varied sizes of pathways, we corrected the values of vector entries as follows [16]:

$$w_{i,j}=(0.5+0.5\times\frac{lw_{i,j}}{\max_{j}lw{}_{i,j}})\times gw_{i}$$

where max*_j_* *lw_i,j_* is the maximum value of all *lw_i,j_* for pathway *j*.

The functional similarity between pathways can be qualified by comparing the vectors. We calculated the functional similarity of a pathway pair, *funSim(p_1_, p_2_)*, by using the cosine measure [17], defined as

$$funSim(p_{1},p_{2})=\frac{\overrightarrow{p_{1}}\cdot\overrightarrow{p_{2}}}{|p_{1}||p_{2}|}$$

The pairwise functional similarity for all pathways was computed.

Hierarchical agglomerative average-linkage clustering with Pearson’s correlation coefficient was performed and visualized by Generalized Association Plots (GAP) [18].
